# Supplementary material for: Functional multispectral optoacoustic tomography imaging of hepatic steatosis development in mice
Source: EMBO Mol Med. 2021 Aug 19;13(9):e13490. doi: 10.15252/emmm.202013490 (PMC8422073; doi:10.15252/emmm.202013490)
Supplement: Supplementary file 2 — Appendix [file EMMM-13-e13490-s002.pdf]

Appendix

Table of Content:

Appendix Figure S1. Comparison of *in vivo* and *ex vivo* spectra of tissues
Appendix Figure S2. Longitudinal monitoring of hepatic ICG clearance in mice
Appendix Figure S3. *Ex vivo* quantification of ICG tracer

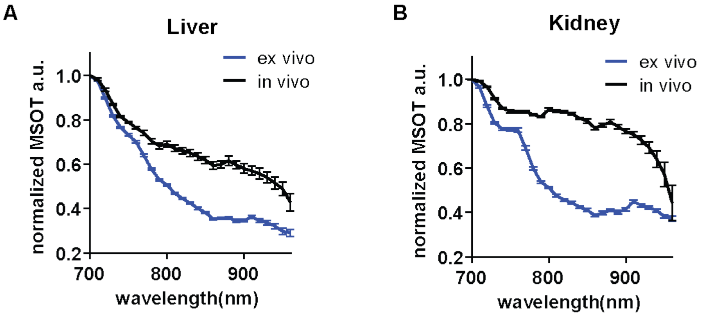

Appendix Figure S1. Comparison of *in vivo* and *ex vivo* spectra of tissues.

A, *In vivo* and *ex vivo* spectra of livers from the same cohort. Data represent the mean (+/- 95% confidence) from 5 animals (n = 5).

B, *In vivo* and *ex vivo* spectra of kidneys from the same cohort. Data represent the mean (+/- 95% confidence) from 5 animals (n = 5).

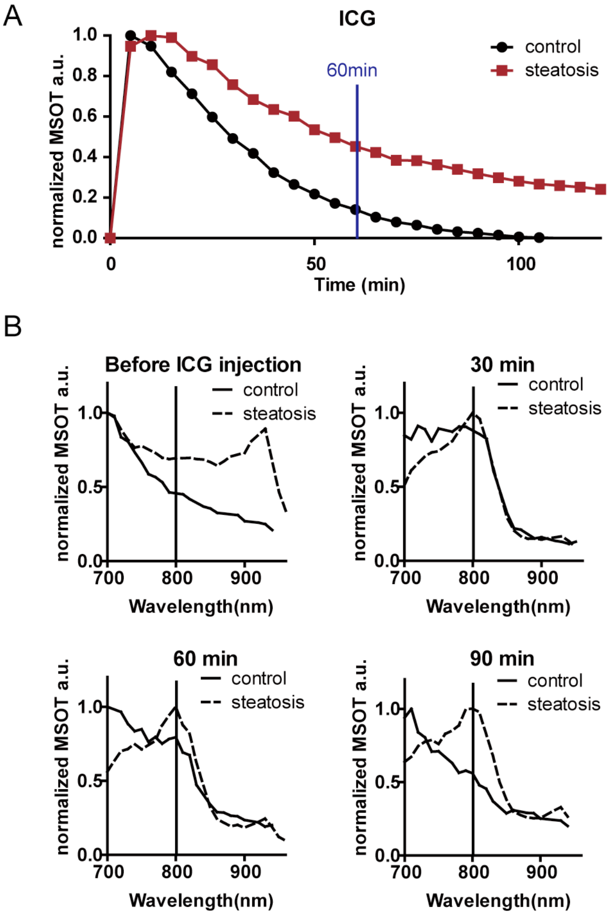

Appendix Figure S2 Longitudinal monitoring of hepatic ICG clearance in mice.

A, 2 hour monitoring of ICG intensity in control and steatotic livers (time interval: 5 minutes). Data expressed the mean from 3 sections in one animal (n = 3). Time point data is normalized to the highest intensity acquired during the observation.

B, Normalized spectra of control and steatotic liver before ICG injection, 30 min, 60 min and 90 min after ICG injection. Data expressed the mean from 3 sections in one animal (n = 3).

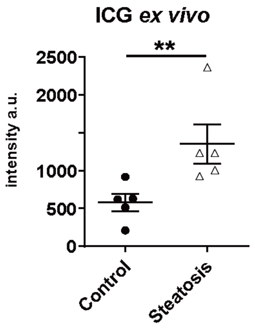

Appendix Figure S3. *Ex vivo* quantification of ICG tracer.

Quantification of mean residual ICG fluorescence intensity of the whole liver tissue section by fluorescence microscopy. Each dot represents data from one animal, in total 5 animals (n = 5). Data represent the mean (+/- 95% confidence). The Mann-Whitney test was used to verify the statistical significance. P = 0.0079.
