# Supplementary material for: Functional multispectral optoacoustic tomography imaging of hepatic steatosis development in mice
Source: EMBO Mol Med. 2021 Aug 19;13(9):e13490. doi: 10.15252/emmm.202013490 (PMC8422073; doi:10.15252/emmm.202013490)
Supplement: Supplementary file 3 — Expanded View Figures PDF [file EMMM-13-e13490-s003.pdf]

Expanded View Figures

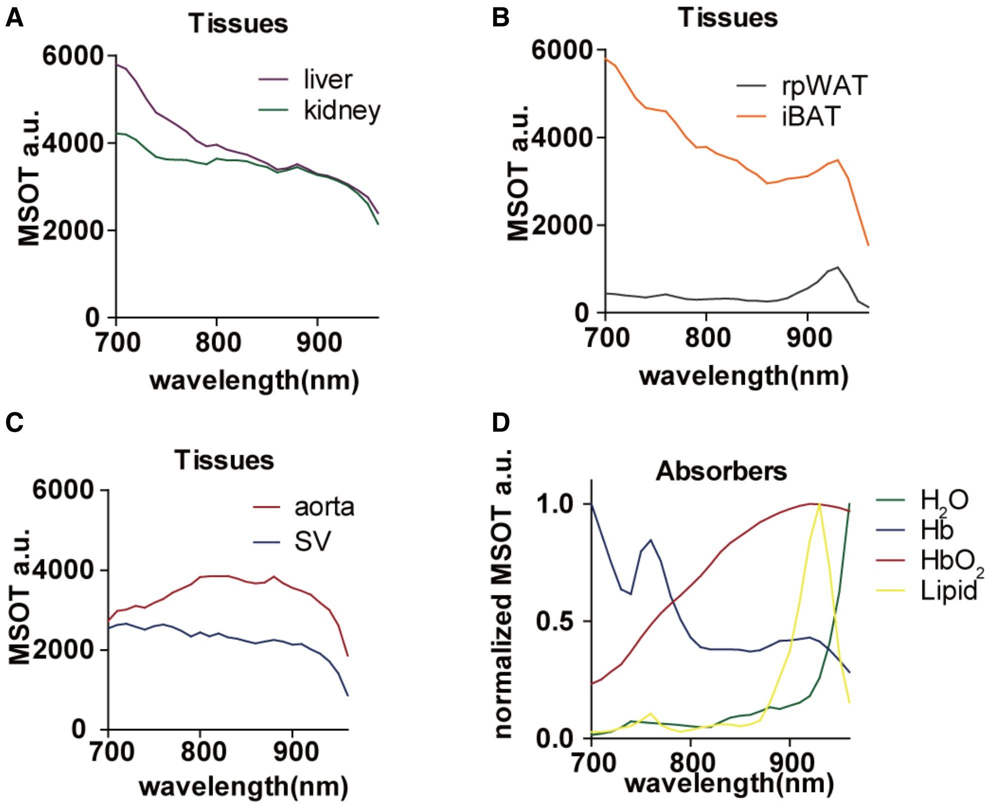

Figure EV1. MSOT spectra of liver, kidney, interscapular brown adipose tissue (iBAT), retroperitoneal white adipose tissue (rpWAT), aorta and Sulzer vein (SV) *in vivo* and main absorbers.

A–C Raw spectra of liver, kidney, iBAT, rpWAT aorta and SV *in vivo*. Data represent the mean from 8 animals (n = 8).

D Normalized spectra of H_2O, Hb, HbO_2 and Lipid. Data are provided as reference in ViewMSOT 3.8 software.

© 2021 The Authors

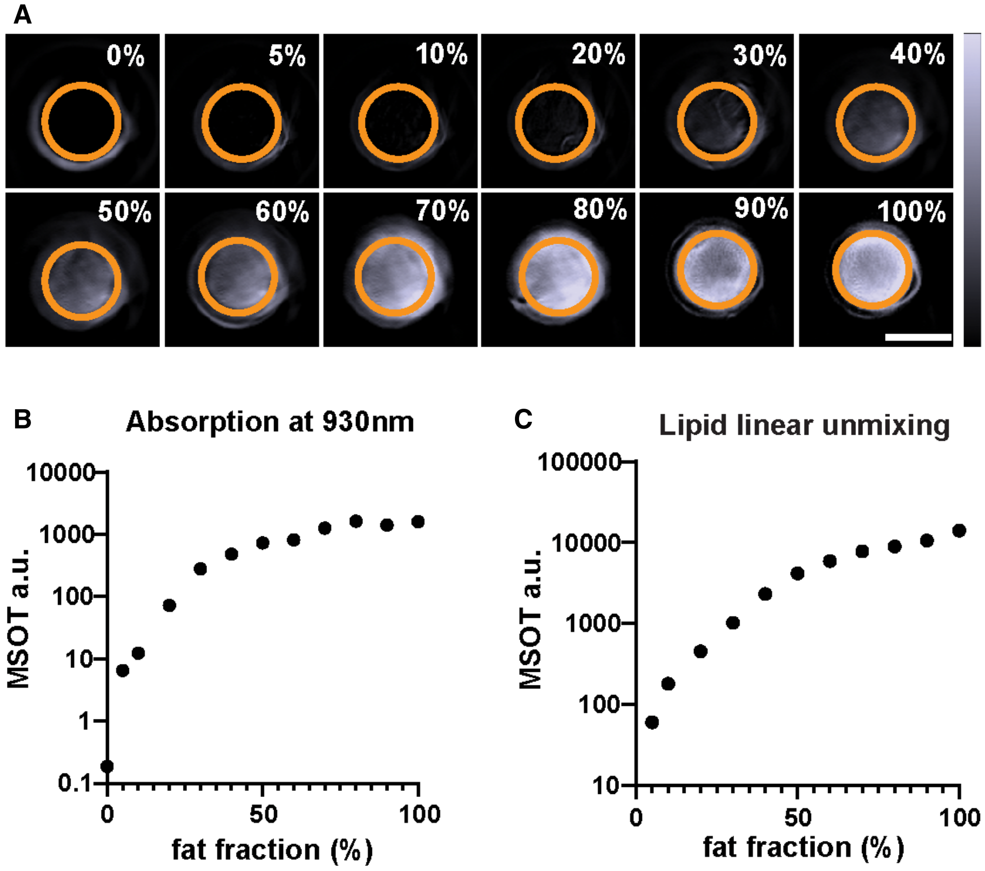

Figure EV2. MSOT imaging of lipid phantoms.

A Reconstructed MSOT images (930 nm) of phantoms containing 0–100% lipid. The lipid fraction and the ROI are indicated on each image. The colour bar shows the colour coding of MSOT a.u. from 0 to 2,000 a.u. (bottom to top). Scale bar: 4 mm.

B Absorption of lipid phantoms at 930 nm. Data represent the mean from 3 sections per phantom.

C Linear unmixing result of lipid from the phantoms. Data represent the mean from 3 sections per phantom.

© 2021 The Authors

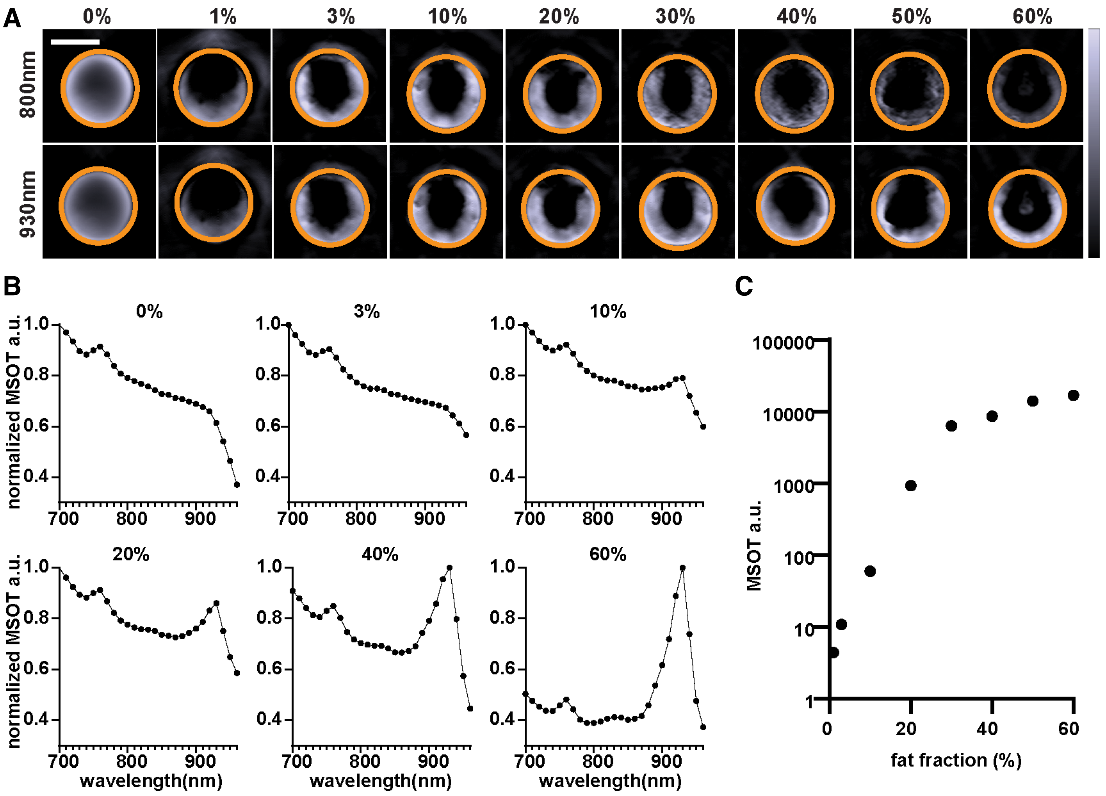

Figure EV3. MSOT imaging of liver–lipid phantoms.

A Reconstructed MSOT images (930 nm) of phantoms contains 0–60% lipid. The lipid fraction and the ROI are indicated on each image. The colour bar shows the colour coding of MSOT a.u. from 0 to 7,000 a.u. (bottom to top). Scale bar: 4mm.

B Normalized spectra of phantoms in A. Data represent the mean from 3 sections per phantom.

C Linear unmixing result of lipid in phantoms. Data represent the mean from 3 sections per phantom.

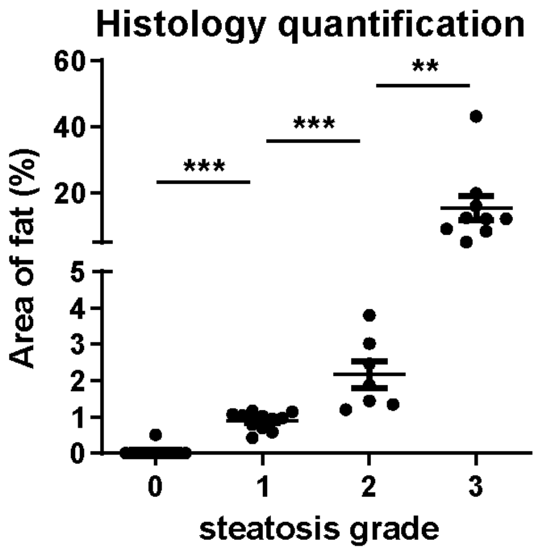

Figure EV4. Histology quantification of lipid in liver.

Each dot represents data from one animal (grade 0: n = 14; grade 1: n = 11; grade 2: n = 7; and grade 3: n = 9). Data represent the mean (± 95% confidence). The unpaired t-test was used to verify the statistical significance. Grade 0 versus grade 1: P = 6.80E-11; grade 1 versus grade 2: P = 0.0007; and grade 2 versus grade 3 P = 0.0086.

© 2021 The Authors

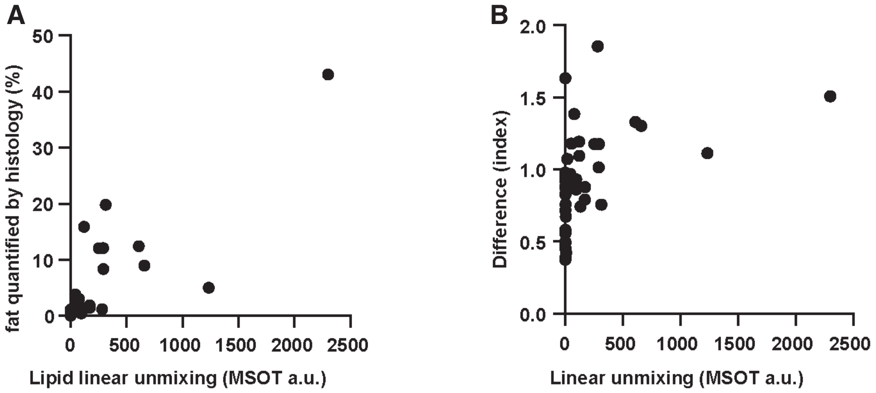

Figure EV5. Correlation analysis of different lipid detection methods.

A Correlation between lipid linear unmixing readout and fat quantification by histology. Each dot represents data from one animal (n = 41).

B Correlation between lipid linear unmixing readout and difference analysis readout. Each dot represents data from one animal (n = 41).
