# Supplementary material for: Functional multispectral optoacoustic tomography imaging of hepatic steatosis development in mice
Source: EMBO Mol Med. 2021 Aug 19;13(9):e13490. doi: 10.15252/emmm.202013490 (PMC8422073; doi:10.15252/emmm.202013490)
Supplement: Supplementary file 4 — Movie EV1 [file EMMM-13-e13490-s001.zip › Movie EV1/Movie EV1.docx]

Movie EV1. Longitudinal ICG tracing in mouse livers by MSOT

The images are focus on lower abdominal region of mice. Time interval: 5 minutes. Total length: 120 minutes. Red: HbO2; blue: Hb; yellow: lipid; green: ICG.
